# Ties that bind: understanding One Health networks and participation for zoonoses prevention and control in India

**DOI:** 10.1186/s42522-024-00118-4

**Published:** 2024-12-01

**Authors:** Festus A. Asaaga, Irfan Shakeer, Aditi Sriram, Kashish Chhotaria, Seshadri Dutta, Darshan Narayanaswamy, Godfred Amankwaa, Mohammed M. Chanda, Subhash L. Hoti, Juliette C. Young, Bethan V. Purse

**Affiliations:** 1 UK Centre for Ecology & Hydrology, Maclean Building, Crowmarsh Gifford, Wallingford, OX10 8BB UK; 2 Ashoka Trust for Research in Ecology and the Environment, Bengaluru, Karnataka 560054 India; 3 One Health Trust, Obeya Pulse, First Floor, 7/1 Halasur Road, Bengaluru, Karnataka 560042 India; 4 ICAR-National Institute of Veterinary Epidemiology and Disease Informatics, Ramagondanahalli, Yelahanka New Town, Bengaluru, Karnataka 560064 India; 5 ICMR-Vector Control Research Centre, Medical Complex, Indira Nagar, Puducherry, 605006 India; 6 Agroécologie, INRAE, Institut Agro, Univ. Bourgogne, Univ. Bourgogne Franche-Comté, Dijon, France

**Keywords:** One health, Zoonosis, Cross-sector collaboration, Stakeholder mapping, India, Low-and middle-income countries

## Abstract

**Background:**

Cross-sectoral collaborations as exemplified by the One Health approach, are widely endorsed as pragmatic avenues for addressing zoonotic diseases, but operationalisation remain limited in low-and-middle income countries (LMICs). Complexities and competing interests and agendas of key stakeholders and the underlying politico-administrative context can all shape outcomes of collaborative arrangements. Evidence is building that organised collaborations are complex political initiatives where different objectives; individual and institutional agendas need to be reconciled to incentivise collaborations.

**Methods:**

Drawing on a qualitative network analysis of published sources on ‘One Health’ stakeholders supplemented with 26 multi-scale (national-state-district level) key-informant interviews (including policymakers, disease managers and public health experts), this paper characterises the fragmented and complex characteristics of institutional networks involved in zoonoses prevention and control in India.

**Results:**

Our results highlight how the local socio-political and institutional contexts interact to modulate how and when collaborations occur (or not), the associated contingencies and stakeholder innovations in circumventing existing barriers (e.g. competing interests, distrust between actors, departmental bureaucracy) to cross-sector collaborations and zoonoses management. Aside from principal actors negotiating common ground in some instance, they also capitalised on political/institutional pressure to subtly ‘manipulate’ their subordinates as a way of fostering collaboration, especially in instances when the institutional and political stakes are high.

**Conclusion:**

Altogether our findings suggest that cross-sectoral collaborations are by-product of political and institutional tinkering as long as individual actors and institutional interests converge and these dynamics must be embraced to embed meaningful and sustainable collaborations in local socio-political and administrative contexts.

## Background

Over the last two decades or so, One Health (OH) collaborations have been gaining momentum in the context of the global and national health security agenda– as a means of marshalling different stakeholders across the human-animal-ecosystems interface to strengthen action for zoonoses research, preparedness, and control [1–5]. The recent Covid-19 pandemic and global monkeypox public health emergencies have given additional impetus for galvanising cross-sectoral response and decision-making on the so-called ‘wicked’ problem of zoonoses which straddles multiple sectors, interests, and geographies [4, 6–8]. The OH approach emphasises the interconnectedness between the health of humans, animals and ecosystems and is widely advocated as a useful concept for creating platforms to strengthen and sustain collaborations between health and non-health departments [9, 10].

Although the notion that cross-sector collaborative working may be valuable for addressing infectious disease problems is not new [11–13], the OH approach (conceptualised on the backdrop of other prior convergence models – e.g. One medicine, planetary health) has gained popularity in public health policy and planning, and transitioned from an approach to a movement [1, 14, 15]. Proponents suggest that the OH approach is ideal for addressing complex societal and health challenges in which different stakeholders operate under different incentive structures [1, 2, 16]. Indeed, the OH literature, specifically in relation to zoonoses, is replete with studies which call for cross-sectoral collaborations, at least for improved zoonoses surveillance and control [1, 4, 17], often identifying the barriers and facilitators of convergence across different socio-political, administrative and cultural contexts see [2, 18, 19].

While exploration of the underlying barriers and facilitators of cross-sectoral collaborations in the existing literature is illuminating, the complex governance structures and relational dynamics that provide the operational contexts for the interactions of multiple stakeholders within and across sectors and associated tensions have yet to be characterised [4, 6, 20, 21]. Several studies show that cross-sectoral partnership arrangements are inherently political [22] and cannot be divorced from the politico-administrative structures that shapes and determines whether incentives for collaborations occur [1, 23]. Thus, the limited understanding of the political economy factors and decision-making processes that drive cross-sectoral collective action has meant that efforts to institutionalise OH collaborations in several LMIC contexts have been at best aspirational [20, 22].

Given the multifactorial drivers and impacts of zoonotic disease outbreaks, they constitute political and economic emergencies that require multi-sectoral collective action transcending the operational mandates of the human and animal health sectors [2, 16, 21, 24]. As such, several OH studies argue for a nuanced and context-specific understanding of existing cross-sectoral networks and conditions for creating and sustaining collaborations [2, 4, 5]. This is particularly true in many LMICs where competing health and developmental priorities may operate to dampen incentives for collective prioritisation and action [3, 20, 25].

To address this research gap, our paper explores the interactions of ‘One Health’ state actors and how they build and maintain networks for key decision-making and action on zoonotic disease control in India. As one of the greatest contributors to the zoonoses burden in South Asia [21, 26, 27], India has yet to implement a national OH policy and/or operational guidelines [21, 28]. Until now, existing literature on OH stakeholders and networks in India has focussed on a subset of cross-departmental networks (agriculture, animal husbandry, human health, environment, and science & technology) at the national level [4], and analysed challenges with OH institutionalisation at the national [2] and city level [21, 28] respectively. We still lack adequate understanding of the complex architecture of cross-scale and cross-organisational networks and partnerships of all actors who are (potentially) involved in zoonoses prevention, preparedness and control in India’s pluralistic governance context. The web of different public and veterinary health institutions for zoonoses management (which falls within the remit of state governments) often with overlapping mandates and interests with respect to disease outbreak prevention and control, necessitates the reconciliation of the different priorities and ties needed to foster a common agenda for effective control [3, 23]. This is particularly important considering the Prime Minister’s Science, Technology, and Innovation Advisory Council (PM-STIAC) recent ratification for the establishment of a National One Health Mission, with the overarching mandate to coordinate, support and integrate all existing OH activities in India [29].

Our empirical investigation therefore addresses the following research questions: (1) what is the current and potential scope of national and state actors and networking activities for tackling zoonotic diseases, (2) what kinds of ties exist across different actors and sectors in terms of zoonotic disease prevention and control, and (3) how can we explain differences, if any, in cross-sector collaborative efforts between national and state level government actors? Drawing on normative stakeholder theory and polycentricity, we characterise the complex, multi-scale ‘One Health’ state actors (in terms of their roles and functions), interrelationships and collaborative networks linked to zoonoses prevention and control. In so doing, we advance the understanding of the configurations of cross-organisational network activities and the contingencies of the ‘ties that bind’ within India’s decentralised health system governance, critical for identifying and implementing sustainable solutions that are adapted to local contexts [21, 23, 30]. Acknowledging the fluidity of the web of negotiated interactions between relevant state actors (in terms of exchange of expertise, information and resources) and the difficulty of capturing same, a blend of document review and semi-structured interviews can offer insights into the complex relational dynamics of actors involved in zoonoses prevention and control in India.

## Methods

A mixed qualitative approach was adopted to better understand the complex stakeholder ecosystem and interactions for zoonoses prevention/preparedness and control in India. Data was thus collected from two principal sources – a scoping review and multi-scale semi-structured interviews with policy decision-makers, disease managers and experts.

### Study system: ‘One Health’ in India

India’s federal character and the decentralised health system governance has meant that zoonoses surveillance and control is nested within and across multiple institutional networks across sectors and scale, resulting in a polycentric system of several actors [2, 3, 31]. Within this context, OH efforts are gaining momentum in India exemplified by pockets of government-initiated activities (in collaboration with inter-governmental agencies (e.g. WHO, FAO, OIE) and international and national non-governmental organisations)) with the view to foster strong cross-sectoral engagement in the surveillance and control of priority zoonotic diseases such as rabies, brucellosis, and foot-and-mouth disease [5, 20]. At the same time, the lack of a national OH policy and guidelines has meant that understanding the scope of existing OH initiatives, the cross-sector collaborative networks and associated contingencies remains critical in guiding the OH institutionalisation process at least in the context of zoonoses prevention and control in India [2, 4, 21].

### Data collection & analysis

Data was collected from two principal sources – a document review and semi-structured interviews with policy decision-makers, disease managers and experts at the national and subnational level in India. In the context of this study, policy decision-makers were defined as the primary decision-makers who directly authorise and inform policy on zoonoses prevention and control or related land/environmental management. OH initiatives comprised activities that receive funding support from government agencies directed at engagement between two or multiple sectors in surveillance and/or control of zoonotic diseases. At the outset of the actor mapping, we carried out a systematic search of the peer-reviewed and grey literature of OH initiatives in India without any time limits. The literature search was conducted using the Web of Science and PubMed databases to identify papers which “One Health” in their titles. The key search terms used were: “one health” OR “collaboration” OR “cross-sectoral collaboration” OR "inter-sectoral collaboration” OR “partnership” OR “actors” OR “stakeholders” AND zoonoses OR zoonosis OR “zoonoses control” OR “zoonoses surveillance AND India). The list of references cited in the collated documents (peer-reviewed papers and reports) led to the identification of additional sources. Following the initial systematic search, a further online search of relevant government ministry websites and repositories was conducted to identify other published policy documents (e.g. cross-sectoral initiatives and annual progress reports, national disease control programmes) at the national and Karnataka state level. The document review was conducted between August 2022 and December 2022 and updated on 5th July 2023.

**Fig. 1:**
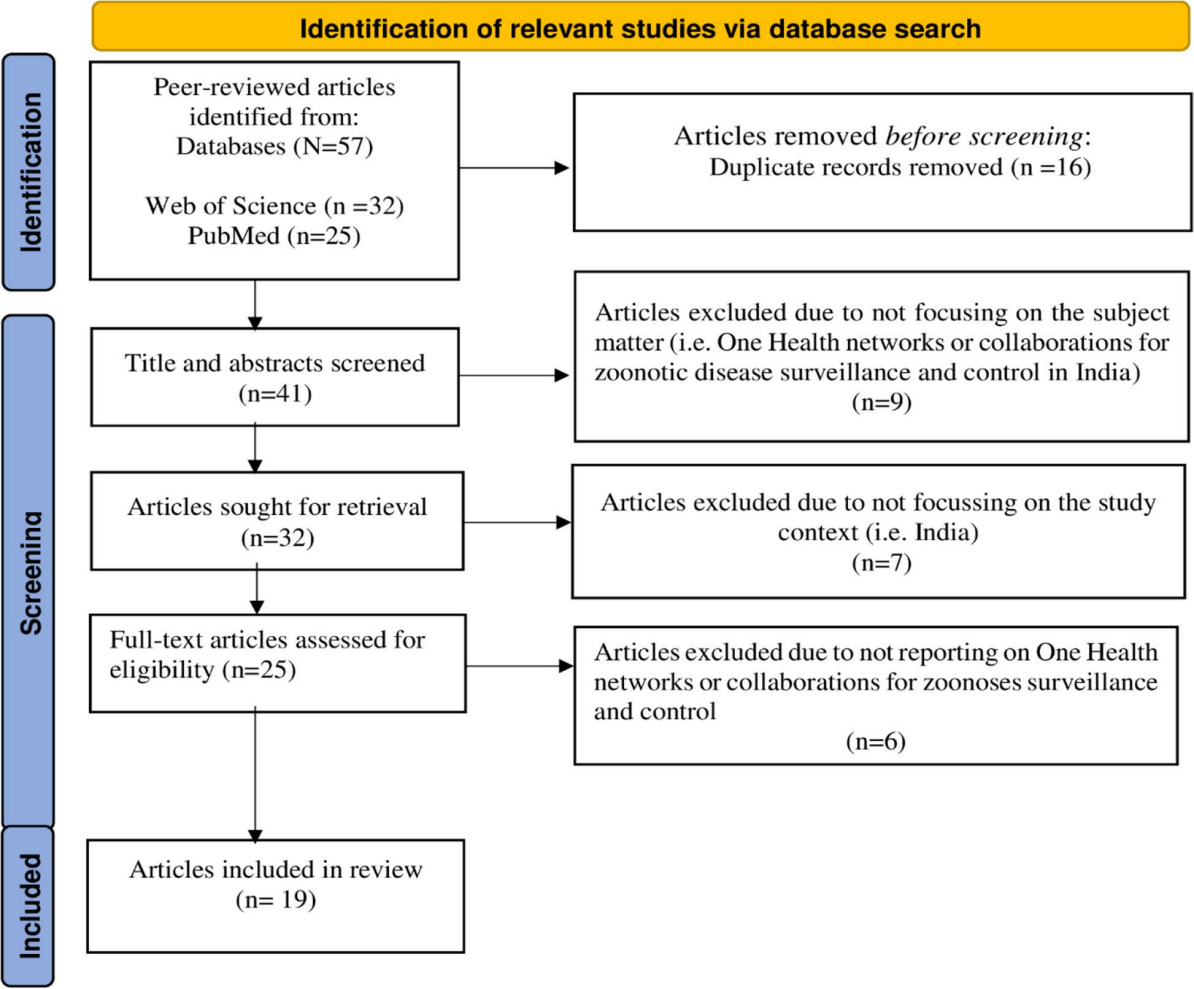
Flowchart of the selection process of relevant peer-reviewed articles focussing on OH activities/collaborations in India.  Adapted from the Preferred Reporting Items for Systematic Review and Meta-Analysis (PRISMA) by Page et al. 2020 [33]

Our systematic search in Web of Science and PubMed databases returned 57 relevant peer-reviewed papers. After title/abstract screening and de-duplication, 32 papers reporting on OH initiatives/ collaborations for zoonoses surveillance and control were retained for full-text review. A total of 13 papers were found to be out of scope of our review. These included papers that did not discuss OH collaborations for zoonoses prevention and control and/or India. 19 papers were finally retained for the actual review (see Fig. 1). The peer-reviewed articles were reviewed by the first author (FA) and the grey documents by three of the co-authors (IF, CK & SD). A pre-designed data extraction form was created and after pilot scoring of 3 academic papers and grey documents, the form was used to extract information on specific OH initiatives/programmes, duration of initiatives, extent of OH engagement undertaken, sectors and actors involved and aims/objectives in respect of different facets of zoonoses management (e.g. surveillance, disease or vector control programmes, outbreak management). Based on the extracted information, we identified actors with decision-making power (i.e. actors directly involved in disease surveillance and outbreak management) and positional actor mapping (i.e. actors in the frontline decision-making whether they take decisions relevant to disease management at the national and state level). These actors were not necessarily involved in the day-to-day implementation or outbreak control. Implementing actors comprised ground level staff who are involved in day-to-day implementation of disease surveillance and outbreak interventions. Representational actors were identified as experts in zoonoses management, and the stakeholder list was validated by key members of the project team who are themselves experts on zoonoses and OH India. An overview map of the OH stakeholder ecosystem was produced to provide a visual illustration of the governance structure and related networks. To validate the stakeholder ecosystem mapping and networks, three experts (from the authors’ professional networks) were consulted: one each from the animal health, human health, and environment sectors respectively. These experts were selected based on their knowledge and involvement in OH activities linked to zoonoses prevention and control in India. A poster presentation of the “One Health” stakeholder ecosystem was presented at the 2022 World One Health Congress in Singapore where further feedback was received [32].

To determine the level of “OHness” in the composition of OH initiatives/programmes mapped see [34], we categorised each OH initiative as intra (i.e. two or more institutions with the same sectoral affiliation), inter (i.e. two or more institutions from two sectors) and mixed (involving multiple institutions from three or more sectors) collaborations. To supplement the literature review and help triangulate the results of the OH stakeholder ecosystem mapping, we conducted semi-structured key-informant interviews with policy decision-makers, disease managers and experts at the national, state (i.e., Karnataka) and district levels. A total of 26 stakeholders were identified across focal organisations involved in zoonoses prevention and control at the national and subnational levels (Table 1). The selection of interviewees was based on the research team’s expert knowledge of the context and OH stakeholder ecosystem mapping under a larger Indo-UK interdisciplinary research project called IndiaZooSystems-Strengthening One Health responses to zoonotic diseases. An overview description of the interviewees who participated is presented in Table 1. All participants were purposively identified and contacted via email and telephone in accordance with their respective institutional protocols. Additional participants were identified through snowball sampling. Most of the interviews were conducted in English and 3 district-level interviews in Kannada, the predominant local language in Karnataka state. Interviews lasted on average 45 min and prior-informed consent was obtained for all interviewees. All the interviews were audio–recorded and transcribed verbatim (into Kannada and English as needed) to avoid information-selection bias.

**Table 1:** List of Interviewees by level of government and sector

Sector/Level	District	State	Federal	Other	Total
Human Health	7	5	2	0	14
Animal Health	0	2	1	0	3
Forestry	1	0	0	0	1
Wildlife	1	1	0	0	2
Academia/ Research	0	0	0	5	5
Non-governmental representatives	0	0	0	1	1
Total	9	8	3	6	26

The collated qualitative data was analysed using a mixture of inductive and deductive approaches following standard procedures for qualitative data analysis [35, 36]. All interview transcripts were imported into NVIVO12 software and coded inductively by two of the authors (AF & AS) independently. We developed a coding framework based on which primary and subthemes were identified and grouped into themes. The collated interview data were anonymised to protect the privacy of participants. An overview of interviewees who participated in the study is presented in Table 1. The study was approved as part of the IndiaZooSystems and IndiaZooRisk + projects by the Health Ministry Screening Committee (HMSC) (VIV0562023) and the respective Institutional Ethics Review Boards of the Ashoka Trust for Research in Ecology and the Environment (IRB/CBC/0003/ATV/07/2018 and IRB/CBC/006/ATV/10/2021) and the Institute of Public Health (IPH) Bangalore (IEC-FR/04/2017) in India, and the Human Research Ethics Committee of the Liverpool School of Tropical Medicine (17–062 and 20–051) in the United Kingdom.

## Results

Our results are presented in three sections: first, we describe India’s ‘One Health’ stakeholder governance structure for zoonoses prevention and control followed by OH initiatives and networks and determinants of stakeholder participation in OH collaborative networks.

### “One Health” stakeholder ecosystem for zoonoses governance

**Fig. 2:**
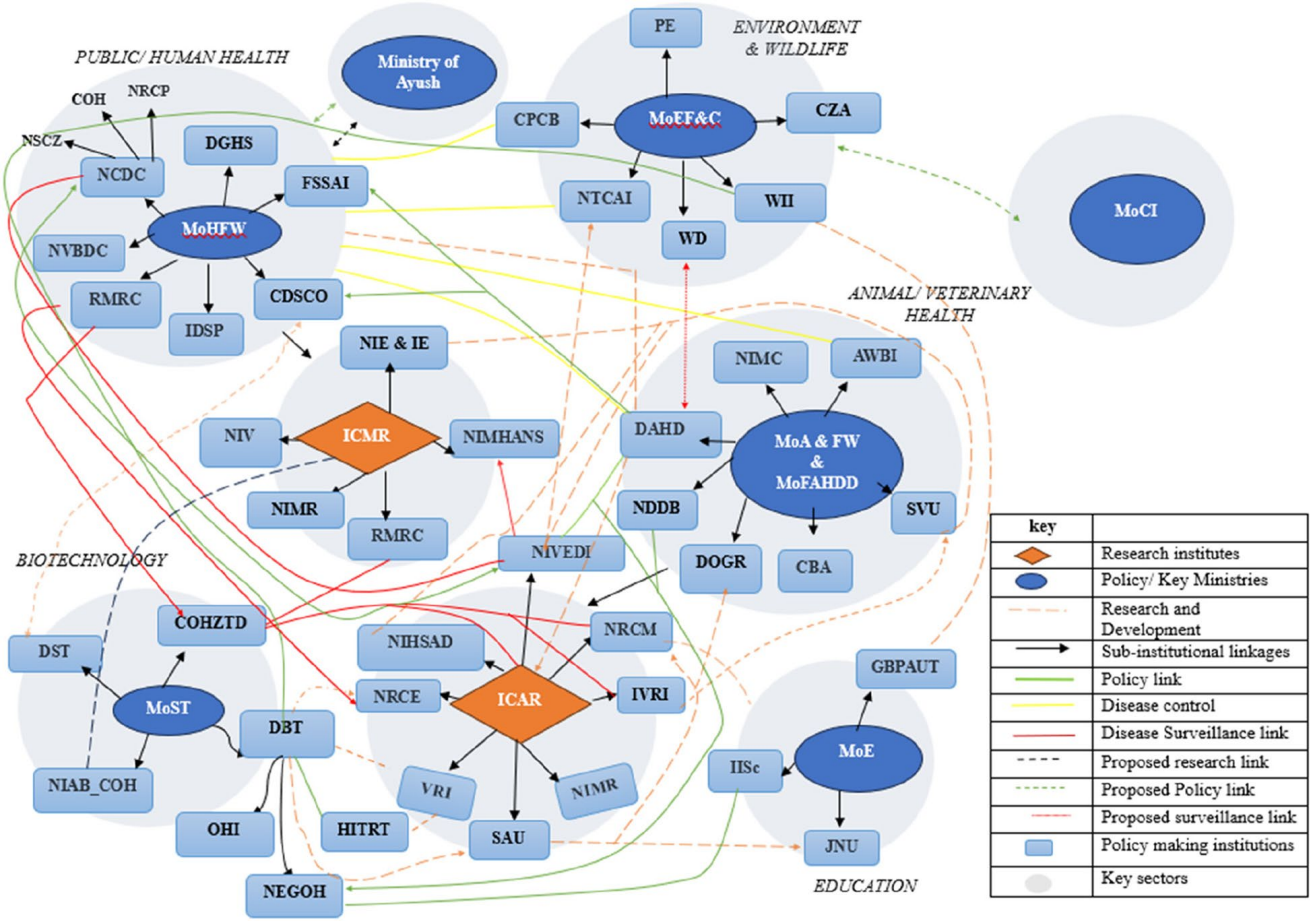
Graphical representation of the state stakeholder ecosystem for zoonotic disease prevention and control at the national level, represented across sectors and their linkages (orange rhombus = research institutes, blue circle = key ministries/policy institutions, light blue rectangle = policymaking institutions, dotted orange lines = research and development links, green line = policy link, yellow line = disease control link, red line = surveillance link, dotted black line = proposed policy line and dotted green line = proposed policy link, grey circle = key sector). ICMR = Indian Council for Medical Research; ICAR = Indian Council for Agricultural Research; NCDC = National Centre for Disease Control; NSCZ = National Standing Committee for Zoonoses; NIE = National Institute of Epidemiology; NIMR = National Institute for Malaria Research; RMRC = Regional Medical Research Centre; AWBI = Animal Welfare Board; DAHD = Department of Animal Husbandry and Dairying; NDDB = National Dairy Development Board; CZA = Central Zoo Authority; PE = Project Elephant;SVU = State Veterinary Universities; DIGR = Directorate of Onion and Garlic Research; MoA&FW = Ministry of Agriculture and Farmers Welfare; NIHSAD = National Institute of High Security Animal Diseases; NIVEDI = National Institute of Veterinary Epidemiology and Disease Informatics; NRCE = National Research Centre for Equines; NRCM = National Research Centre on Meat; SAU = State Agricultural Universities; TFZ = Task Force on Zoonoses; DBT = Department of Biotechnology; DST = Department of Science and Technology; COHZTD = Consortium for One Health to Address Zoonotic and Transboundary Diseases; HITRT = Haffkine Institute for Training, Research and Testing; NEGOH = National Expert Group on One Health; NIAD_COH = national Institute of Animal Biotechnology Centre for One Health; IISC:GBPUAT = Govind Ballabh Pant University of Agriculture and Technology; Indian Institute of Science; JNU = Jawaharlal Nehru University; MoEF&C-CPCB = Central Pollution Control Board, Ministry of Environment, Forestry and Climate Change; NTCAI = National Tiger Conservation Authority of India; WD = Wildlife Department; WII = Wildlife Institute of India; CDSCO = Central Drugs Standard Control Organisation

**Fig. 3:**
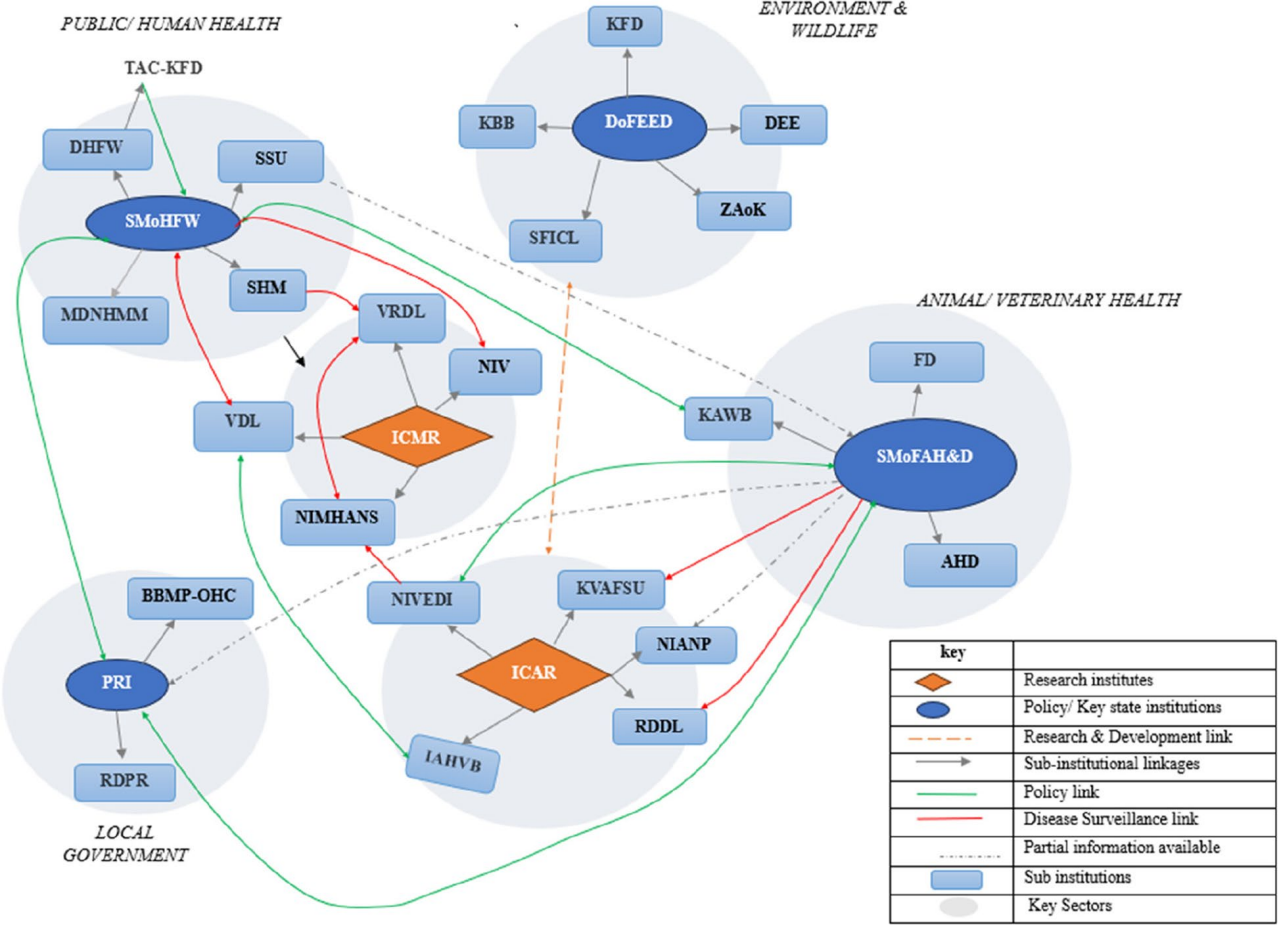
Graphical representation of the Karnataka state stakeholder ecosystem for zoonotic disease prevention and control represented across state sectors and their linkages (orange rhombus = research institutes, blue circle = key state ministries/ policy institutions, grey circle = key sectors, light blue rectangle = policy making state institutions; dotted orange lines = research and development links, green line = policy link, yellow line = disease control link red lines = surveillance link, dotted black lines = proposed policy link and dotted green lines = proposed policy linkage). ICMR = refers to Indian Council for Medical Research; ICAR = Indian Council for Agricultural Research; NIV = National Institute of Virology; SMoH&FW = State Ministry of Health and Family Welfare; DHFW = Department of Health and Family Welfare; NIMHANS = National Institute of Mental Health and Neurosciences; SHM = State Health Mission; SSU = State Surveillance Unit; TAC-KFD = Technical Advisory Committee on Kyasanur Forest Disease; VDL = Virus Diagnostic Laboratory; SMoFAH&D = State Ministry of Fisheries, Animal Husbandry and Dairying; BBMP-OHC = Bruhat Bengaluru Mahanagara Palike-One Health Cell, PRI = Panchayati Raj Institutions; NIANP = National Institute of Animal Nutrition and Physiology;; KBB = Karnataka Biodiversity Board; KFD = Karnataka Forest Department; DoFEED = Department of Forest, Ecology and Environment Development; SSU = State Surveillance Unit; DEE—Department of Ecology and Environment; SHM = State Health Mission; VRDL = Virus Research and Diagnostic Laboratory; ZAoK = Zoo Authority of Karnataka; AHD = Animal Husbandry Department; RDDL = Regional Disease Diagnostic Laboratory; RDPR = Rural Development and Panchayat Raj Department; KSFIC = Karnataka State Forest Industries Corporation Limited

Given the pluralistic governance structure (comprising different state actors and institutions operating at multiple levels and sectors) for zoonoses surveillance and control in India [2], it was important to map the cross-sectoral landscape of current OH human, animal and environmental health institutions at the national and state level. Figures 2 and 3 represent an overview of the stakeholder landscape and their ties/linkages (illustrating how information was exchanged through the network) regarding zoonoses prevention and control. Of the 57 national level institutions involved in zoonoses surveillance and control across all the key sectors (see grey circles), 10 played a central/primary sectoral role in disease surveillance and detection.

Whereas this landscape assessment was not exhaustive, as it was not possible to include every institution/organisation (particularly non-governmental organisations) contributing to zoonoses management, the mapping exercise revealed that there was a high degree of intra-institutional interaction across the policy network (policymaking, disease surveillance and control) between different government and quasi-government institutions. The qualitative interviews highlighted the complexity of zoonoses governance as different institutions are placed under different administrative structures posing risk to the alignment and level of cross-sectoral engagement, particularly outside of outbreak situations. Several informants attributed the predominance of human health institutions to the lack of shared sense of responsibility for zoonotic disease management whilst others elaborated how the extant landscape of institutions were somewhat exclusionary of representation of environment-affiliated institutions (see Sect. " Perceived legitimacy, authority and representation"). Comparing the national and state level mapping, one striking observation is that the majority of the ICMR and ICAR affiliated institutions were involved in a largely research-based network on disease surveillance initiatives. NIVEDI, NRCM, COHZTD and NIE were the most well-connected research-oriented institutions with respect to zoonoses surveillance and control initiatives. In terms of policy-based networks on disease surveillance, key institutions such as the MoH&FW, NSCZ, MoFAH&D and DBT were the most well-connected. Altogether, Figs. 2 and 3 give the indication that collaborations were mostly mandate driven and often disjointed, with peripheral actors less likely to be involved in extant OH networks on zoonoses surveillance and control.

### “One Health” initiatives and networks for zoonoses prevention and control

Table 2 summarises current OH collaborative initiatives that have been operationalised and their defining characteristics (including focus, stakeholders’ representation, collaboration type and diseases of interest). Overall, 23 OH relevant initiatives on zoonoses surveillance and control were identified, revolving around specific research themes including outbreak emergencies (*n* = 12 initiatives) and awareness raising and capacity building (*n* = 11). Between 2020 and 2023, during the Covid-19 pandemic, 7 OH-related initiatives were created (at the central and state level), including the One Health Commission for surveillance. Of the OH initiatives mapped, 7 were operationalised at the national level and mostly short-term and programme-based (as they emanated from time-bound research projects), 6 were active and the remainder did not provide any information as to their status.

**Table 2:** Overview of one health initiatives for zoonoses management in India

OH initiative/programme	Aim/focus	Year	Focal disease	Stakeholders	Type of initiative	Form	Scale	Scope	Sectoral affiliation
Animal Pandemic Preparedness Initiative (APPI)	Enhance India's preparedness and response to animal pandemics, with a focus on zoonotic diseases that pose a threat to both animal and human health	2023 – present	Zoonoses	• Department of Animal Husbandry and Dairying • Ministry of Fisheries, Animal Husbandry & Dairying • Research institutes of ICAR and ICMR • World Health Organisation • Food and Agriculture Organisation	Disease outbreaks prevention and control (Animal pandemic preparedness)	Inter	National and sub-national	Programme implementation (research and capacity building)	AH & PH
Animal Health Systems Support for One Health (AHSSOH)	Create an ecosystem for a better animal health management system using the One Health approach covering five (05) states in India	2023—present	Zoonoses	• Department of Animal Husbandry and Dairying • Ministry of Fisheries, Animal Husbandry & Dairying • Research institutes of ICAR and ICMR • World Bank • World Health Organisation • Food and Agriculture Organisation	Disease outbreaks prevention and control (Animal pandemic preparedness)	Inter	National and sub-national	Programme implementation (Solution-based)	AH & PH
National One Health programme for Prevention and Control of Zoonoses (NOHP-PCZ)	Operationalise “One Health” Mechanisms for prevention and control of Zoonoses through strengthening inter-sectoral coordination among all stakeholders at the national, state, and district and sub-district levels	2017–2026	Priority zoonoses	• Centre for One Health (National Centre for Disease Control) • Department of Animal Husbandry and Dairying • Wildlife Institute of India • Civil Society organisations	Disease outbreak prevention and control	Mixed	National and sub-national	Programme implementation (Solution-based)	PH, AH & Wildlife
One Health Support Unit under Department of Animal Husbandry and Dairying	Support the Government of India in developing India’s National One Health Platform		Zoonoses	• Department of Animal Husbandry and Dairying • Ministry of Fisheries, Animal Husbandry & Dairying • Confederation of Indian Industry • Bill & Melinda Gates Foundation	Disease outbreak prevention and control	Mixed	National	Programme implementation (Solution-based)	PH, AH & Wildlife
One Health programme on highly pathogenic emerging diseases in Asia	Advocacy and operationalisation of OH at regional and country levels in the Asia–Pacific region for avian influenza prevention and control	2009–2014	Avian Influenza	• National Centre for Disease Control • Indian Council of Medical Research • Indian Council of Agricultural Research • World Health Organisation	Research development collaboration	Mixed	International	Agreement (Level-based- research)	PH & AH
Programme for Inter-sectoral Coordination for Prevention and Control of Zoonotic Diseases	Foster inter-sectoral collaboration for prevention and control of zoonotic diseases of public health importance	2012- present	Priority zoonoses (rabies, anthrax, scrub typhus, leptospirosis)	• Ministry of Health and Family Welfare (MoH&FW) • Ministry of Agriculture and Farmer Welfare • Wildlife Institute of India	Disease outbreaks and prevention and control	Inter	National and sub-national	Programme implementation (Solution-based)	PH, AH & Wildlife
One Health Roadmap (OHR)	Tackle the most urgent health threats		Zoonoses	• Department of Biotechnology	Research development collaboration	Inter	National	Agreement (Third-party based)	Biotechnology
Roadmap to combat zoonoses in India (RCZI) Initiative	Assessing research options for controlling zoonoses in India	2010–2015	Zoonoses	• Public Health Foundation India • Civil Society organisations	Research and laboratory collaboration	Mixed	National	Agreement (Research development)	PH
Rabies Control Initiative – Tamil Nadu	Rabies prevention at a sub-national level (Tamil Nadu)	2004	Rabies	• Directorate of Public Health & Preventive Medicine • Directorate of Education • Directorate of Rural Health & Medical Services • Municipal Administration Department • Department of Animal Husbandry and Dairying • Tamil Nadu Medical Services Corporation • Civil Society organisations	Disease outbreaks prevention and control	Mixed	Sub-national (Tamil Nadu)	Programme implementation (Level-based (Population, individual)	PH, AH, education & welfare
The Sikkim Anti-Rabies and Animal Health (SARAH) program	One Health model of sustainable dog-mediated rabies elimination	2006–2015	Rabies	• Government of Sikkim • Vets Beyond Borders • Fondation Brigitte Bardot	Disease outbreaks prevention and control	Inter	Sub-national (district)	Programme implementation (Solution-based)	AH
One Health Collaborative Action Strategy for Control of KFD (Wayanad)	Control the 2015 Kyasanur Forest Disease outbreak (Wayanad)	2015	Kyasanur Forest Disease	• District Medical Officer • District Animal Husbandry Officer • District Forest Officer-North/South • Veterinary University • Tribal Development Officer • District Police Officer • Media	Disease outbreaks prevention and control	Mixed	Sub-national	Programme implementation (Solution-based)	PH, AH, welfare
Tamil Nadu Veterinary & Animal Sciences University (TANUVAS) and Tamil Nadu Dr MGR Medical University (TNMGRMU) MOU	Joint academic and research activities (including trial for a SARS-CoV2 vaccine candidate developed by TNMGRMU	2020–2020	Covid-19	• Tamil Nadu Veterinary and Animal Sciences University • The Tamil Nadu Dr MGR Medical University	Research and laboratory collaboration	Inter	Subnational	Programme implementation (Level-based research)	PH & AH
Guru Angad Dev Veterinary and Animal Sciences University (GADVASU) and Dayanand Medical College and Hospital, Ludhiana	MOU for testing samples for Brucella spp.		Brucellosis	• Guru Angad Dev Veterinary and Animal Sciences University (GADVASU) • Dayanand Medical College and Hospital, Ludhiana	Research and laboratory collaboration	Inter	Subnational	Agreement (Level-based- research)	PH & AH
TANUVAS and Tamil Nadu State Public Health Department	MOU on testing and sharing results on leptospirosis	2020-present	Leptospirosis	TANUVAS Tamil Nadu State Public Health Department	Research and laboratory collaboration	Inter	Subnational	Agreement (Level-based research)	PH & AH
Indian Council of Medical Research (ICMR)-National Institute of Virology, Pune for KFD and CCHF outbreaks in India	One Health capacity building and development of laboratory networks for rapid diagnosis and management of Kyasanur Forest Diseases and Crimean Congo Haemorrhagic fever (CCHF) outbreaks	2018	Kyasanur Forest Disease and Crimean Congo Haemorrhagic Fever	• National Institute of Virology • State Health Department • District Administration	Disease outbreaks prevention and control	Inter	Subnational	Programme implementation (Solution-based)	PH
MoH&FW, Directorate of Health Research, Indian Council of Agricultural Research (ICAR), State Health Department, State Animal Husbandry, and District Administration for the outbreak of Nipah virus	Control 2018 Nipah outbreak in the state of Kerala	2018	Nipah virus	• MoH&FW, Directorate of Health Research • Indian Council of Agricultural Research (ICAR) • State Health Department • State Animal Husbandry • District Administration	Disease outbreaks prevention and control	Inter	Subnational	Programme implementation (solution-based)	PH & AH
Veterinarians deployed in isolation wards of hospitals in Haryana state through CO-JEET-victory over Covid-19 extending support to medical personnels	Setting up of Covid-19 facilities to support control		Covid-19	• Animal Husbandry Department • Veterinary Corps – Armed Forces • Medical hospitals	Disease outbreaks prevention and control	Inter	Subnational	Programme implementation (solution-based)	PH & AH
GADVASU and Government Medical colleges, Amritsar and Patiala	Training on use of real-time PCR diagnostic testing	2020–2020	Covid-19	• GADVASU • Government Medical colleges (Amritsar and Patiala)	Disease outbreaks and control	Inter	Subnational	Programme implementation (Solution-based)	PH & AH
TANUVAS and Kings Institute of Preventive Medicine and Council of Scientific and Industrial Research-Center for Cellular and Molecular Biology	Collaborate to sequence 21 complete genomes of SARS-CoV2	2020–2020	Covid-19	• TANUVAS, Kings Institute of Preventive Medicine • Council of Scientific and Industrial Research-Center for Cellular and Molecular Biology	Disease outbreaks prevention and control	Inter	Subnational (State)	Programme implementation (Solution-based)	PH & AH
GADVASU and the State health department	Awareness raising on symptoms and preventive measures for prevalent diseases such as brucellosis	2016–2016	Brucellosis	• GADVASU • Punjab Agricultural University • State Health Department	Disease prevention	Inter	Subnational (Punjab)	Programme implementation (Level-based (population)	PH & AH
One Health Consortium for surveillance of bacterial and viral infections	Development of additional diagnostic methodologies for the surveillance and understanding the spread of emerging diseases	2021—present	Bacterial, viral and parasitic infections of zoonotic and transboundary pathogens	• Department of Biotechnology (DBT)—National Institute of Animal Biotechnology Hyderabad • AIIMS • IVRI • GADVASU • TANUVAS • MAFSU • Assam agricultural and veterinary university • ICAR, ICMR centres • Wildlife agencies	Disease surveillance and control	Mixed	National	Programme implementation (Solution-based)	PH, AH, wildlife, forestry & welfare
OH approach being utilised for the control of anthrax in tribal villages of Odisha involving healthcare sector, animal care sector, NGOs and community social welfare groups	Control of anthrax in a tribal district of Odisha		Anthrax	• Community social welfare groups • Clinical service providers • Veterinary doctors • Forest guards (animal care sector)	Disease outbreaks prevention and control	Mixed	Subnational	Programme implementation (Solution-based)	PH, AH, forestry & welfare
OH centre for established by GADVASU engaging communities in training and awareness programs related to zoonoses, AMR, food and water sanitation and biosecurity measures	Community outreach and education about zoonotic diseases	2016	Zoonoses	• GADVASU • Affected communities	Community outreach and engagement	Inter	Subnational	Programme implementation (Solution-based)	AH & welfare

*PH* Human/Public health, *AH* Animal health

Table 2 highlights some marked differences in the distribution of resources and disease focus of current OH-related initiatives linked to zoonoses surveillance and control. Covid-19, rabies and avian influenza were the topmost diseases of focus, reflecting their high status on the existing NCDC zoonoses prioritisation list [3]. The conspicuous focus on rabies for example, relative to other endemic high burden zoonoses (e.g. KFD, scrub typhus, leptospirosis) in current OH initiatives is unsurprising considering the groundswell of international funding support and high global and national policy prioritisation of rabies (e.g. Global Alliance for Rabies Control, the Sikkim Anti-Rabies and Animal Health (SARAH) program, ASEAN rabies meeting) on account of the high rabies burden in humans and animals in India [37, 38]. This finding is broadly consistent with previous research suggesting the characterisation of rabies as a global health security priority derives from the common-placed perception that preventable zoonoses (such as rabies) are more realistic/attainable policy foci (relative to other lesser-known diseases) considering the high potential for disease elimination and high human and animal cost of mortality [3, 39]. This suggest that the funding regime dictates which diseases will be prioritised for cross-sector (e.g. human and animal health collaboration) surveillance and/or control. Synonymous with other global south contexts, current Indian OH initiatives linked to zoonoses management are characterised by limited community engagement/involvement [34]. It thus follows that for equitable OH representation on zoonoses governance, the participation of affected communities remains paramount in the agenda setting [34, 40].

### Stakeholder participation in one health networks

Current OH networks/initiatives for zoonoses management are largely sectorally driven and dominated by human and animal health affiliated institutions, with limited involvement of non-health institutions (e.g. wildlife) (Table 2). The network structure of OH initiatives also suggests homophily (self-selecting actors that know each other), as the most collaborated stakeholders are affiliated to the same department and/or sector with previous history of engagement. For instance, the ICMR and ICAR affiliated institutions collaborated in most of the networks on zoonoses surveillance and research. The qualitative interviews supported this observation as a common refrain following the acknowledgement of the importance of cross-sectoral engagement was that familiarity and past experiences were key levers driving the nature of OH collaborations. In this respect, both animal and human health interviewees at the national level mentioned that large-scale and nation-wide nature of ICAR and ICMR provided an avenue to successfully collaborate in combating disease emergencies and epidemics. Typical views expressed by two high ranking officials (from two of the most collaborated institutions) in separate interviews were as follows:

“*OneHealth concept itself is moving around among the researchers only and moving around the like you know, universities only. So far, we are not able to convey the message to the people who work on the ground*” (Interview 2, Public Health-State level).

“*So with the different agencies, we do have coordination but now we want to take this one health concept forward with the tighter integration with veterinary and the health department. But we want to do that. So that's our vision for the probably the next few years that will do*” (Interview 7, Public Health-National level).

OH networks showed limited or no direct community engagement corroborating the argument that current OH initiatives lacked community focus [34]. For example, the flagship 27 agency driven consortium (i.e. The One Health consortium) conspicuously lacked any detail on community engagement. These networks provided scant information on the financial arrangements of OH networks, which is critical determinant on their sustainability in the long run.

### Drivers of cross-sector “OneHealth” networks – marriage of necessity or convenience?

**Fig. 4:**
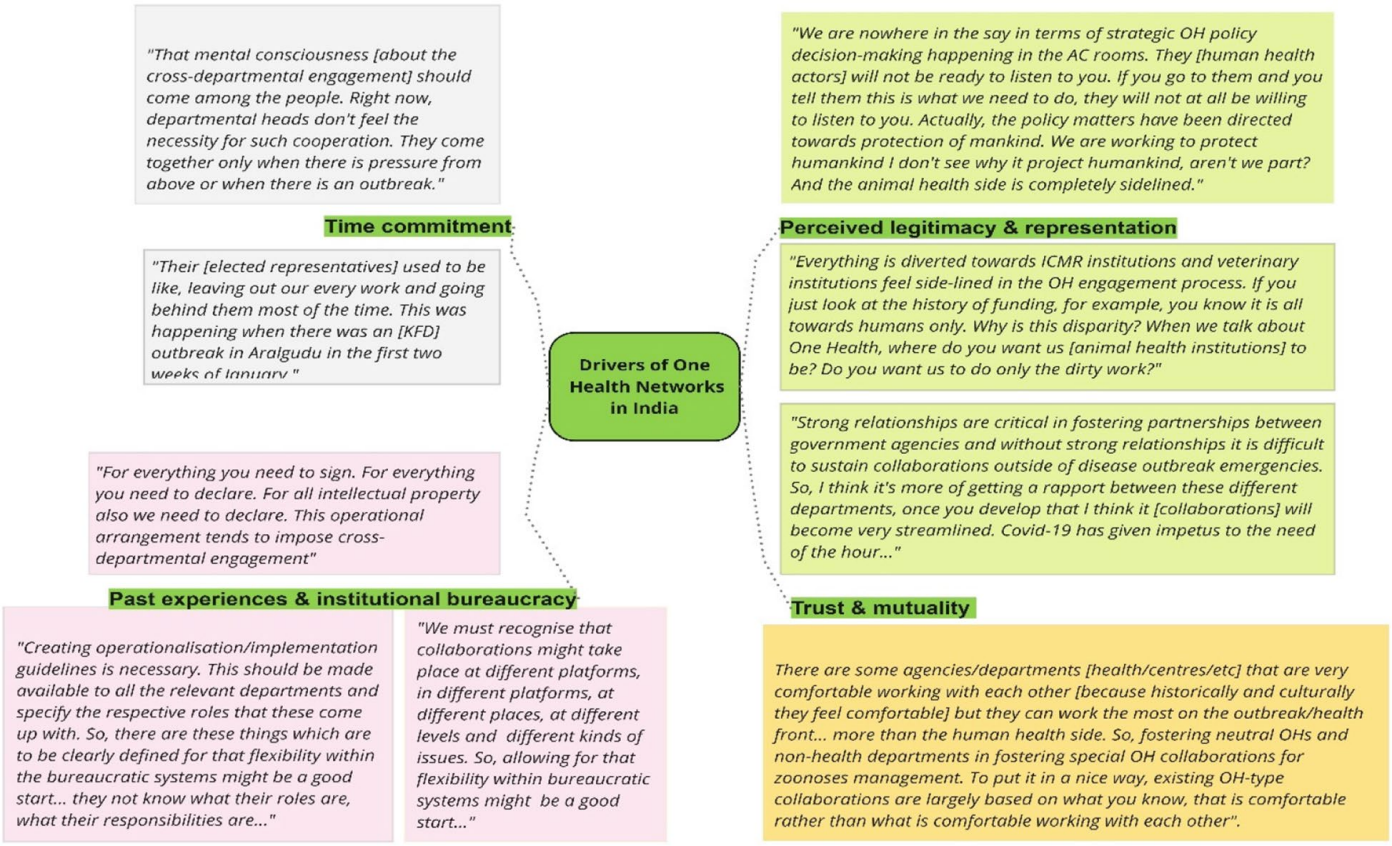
Visual representation of the factors influencing cross-sector engagement for zoonoses management: specific drivers of OH networks (green boxes); with exemplar quotations in different colours (orange, pink, light green and grey respectively)

As shown in Fig. 4, several interacting factors shaped cross-sectoral actors’ motivation to collaborate or not. Prominent themes emerging from the multi-scale interviews were time commitment, past experiences of engagement, bureaucracy, authority and representation, trust and mutuality. Each is discussed in turn.

#### Time commitment

A recurrent theme across the interviews was the lack of time as a limiting factor in stakeholder involvement in OH networks. A number of district level actors (responsible for programme implementation) in particular observed that time constraint is a key feature of their jobs as they had more immediate tasks to attend to as opposed to what they perceived as ‘tangential tasks’. Indeed, some interviewees observed this as a ‘zero sum’ relationship between existing priorities and collaborative engagements, arguing that for one aspect to improve the other must suffer. In the existing institutional environment, for example, some non-health actors interviewed hinted that efforts to drive more collaborative engagements (particularly outside of their core mandates) tended to result in reduced time for their core departmental obligations which was a strong dis-incentive to cross-departmental engagement. Relatedly, some participants also expressed concern that close collaboration risks masking/obscuring the visibility and/or contributions of other fringe (mostly non-health) departments thereby entrenching the hegemony dominance of the public health departments (Interview 6, Animal Health-State level). Two typical explanations proffered by an animal health actor and a human health colleague in their respective interviews are instructive:

“*Right now departmental heads don’t feel the necessity for such cross-departmental as there is no dedicated budget to support such cross-departmental engagements*. *They [departmental heads] only come together when there is pressure from above or when there is an [disease] outbreak emergency* (Interview13, Animal Health-District level).

“*Respective departments* [*placed under different administrative structures*] *have their own priorities so staff are overloaded and do not have time, energy and resources to engage other sectors, except during disease emergencies. This may be one of the reasons why there is limited cross-departmental exchange. Otherwise, technocrats want to work together. This situation may not necessarily be only in India.” (*Interview 9, Public Health-State level).

This view was also echoed by other senior human health civil servants (operating at the state and national levels respectively) who argued that the time constraints particularly on the part of implementing actors at the district level has meant that cross-departmental collaborations typically occur during outbreak events (out of necessity) because of “*external pressure*” from elected representatives exerted through more senior colleagues in the bureaucratic chain. A case in point, as explained by district-level disease manager, is the widely reported Kyasanur Forest Disease outbreak in Aralgudu (Karnataka state) where alleged high public concern hysteria (following media hype of the outbreak) coincident with local *panchayat* elections led the local-level elected representatives to mount political pressure on their hierarchy, which culminated in “*things moving very fast*” in terms of coordinated cross-departmental response (Interview 21, Public Health-District level). A typical perspective by a district level official on the merits of such externally motivated/inspired cross-departmental engagement is illuminating:

“*Their [elected representatives] used to be like leaving out our every work and going behind them most of the time. This was happening when there was KFD outbreak in Aralgudu in the first two weeks of January* [2018]*. So that is one issue but still if you think in one way, it gave good effect because of media hype, things started moving very smoothly and fast.*” (Interview 22, Public Health-District level).

Acknowledging the institutional needs and, in some instance, conflicting priorities, several participants favoured a less prescriptive (i.e. loose) approach to cross-departmental engagement as that afforded the latitude for officials to determine who they collaborate with and the collaboration type as aligned with their own departmental priorities. The foregoing also finds expression in Ribeiro et al.’s [41] argument that cross-sector OH collaborations are often problematic to the extent multiple actors tended to interpret OH within the context of their [departmental] mandate and activities.

#### Past experiences and institutional bureaucracy

A general consensus across the interviews was that past experiences and the regulatory environment were critical determining factors that shaped the outcomes of cross-sector collaborations for zoonoses surveillance and control. When asked about the role of institutional context in driving collaborations, a number of interviewees (mostly at the district level) noted that the administrative requirements did impact significantly on cross-departmental engagements. Participants highlighted that the top-down bureaucratic structure has meant that state level agencies outside of outbreak emergencies was problematic. An animal health official for instance remarked, *“for everything you need to sign! For everything you need to declare! This operational arrangement tends to impede cross departmental engagement”* (Interview 3, Animal Health-State level). Although the majority of senior level interviewees (at the state and national levels) did not explicitly acknowledge that institutional bureaucracies adversely impacted cross-sector collaborations, a cross-section of them implicitly conceded that in the absence of clear operational guidelines, the somewhat “siloed” institutional design and bureaucratic behaviour shaped the general posture to cross-sector collaborations. In fact, a district-level wildlife official concurred that they sometimes assumed a rather lethargic/hesitant posture, if from their standpoint, proposed initiatives did not align with their own departmental budget priorities. Two state-level informants reflecting on the prevailing institutional context had this to say:

*“Creating operationalisation guidelines is necessary. This should be available in all the relevant departments and specify the respective roles in disease control efforts as these things may not be a priority in other (non-health) departments so they may not know what their roles are, what their responsibilities are…”* (Interview 9, Public Health-State level).

*“We must recognise that collaborations might take place at different platforms, in different places, at different levels and different kinds of issues. So, allowing for that flexibility within bureaucratic systems might be a good start…”* (Interview 4, Wildlife-State level).

On a differing perspective, a few participants argued that despite the institutional bureaucracy, front-line personnel have successfully negotiated cross-sector collaborative opportunities (leveraging their professional goodwill, informal personal networks and prior experiences where necessary) when the exigencies of the times necessitated same. To further buttress this assertion, a few national policy decision-makers cited the ‘Covid-19’ coordinated response as a case in point, depicting the inherent negotiability of the administrative structures supporting collaboration in instances where departmental priorities/goals align. A high ranking official at the NCDC, for instance, disclosed that a *“good amount of funding has been allocated for OH-related activities. Especially after this Covid-19 pandemic, everybody has realised it* [i.e. value of cross-sector engagement] *and many other stakeholders ICMR-affiliated institutions. You must have heard about the National One Health Project that has been established”* (Interview 7, Public Health-National level). In furtherance to this, another national-level official highlighted that the past experiences (e.g. Nipah outbreaks) provided a good window of opportunity in terms of rich institutional memory that could always be leveraged in driving future cross-sector collaborations:

*“Nipah is one of the important disease which has made us learn so many things in terms of collaboration with you know, wildlife sector and the animal husbandry. So now there is a positive move towards the wildlife and the animal husbandry sectors as we are now coming together and having a constructive dialogue across the sectors”* (Interview 10, Public Health-National level).

The foregoing was also tempered by the frequent rotation, staff attrition and turnover which has meant that it is often difficult to retain institutional memory for building lasting cross-institutional relationships even if backed by strong political endorsement at the national level.

#### Perceived legitimacy, authority and representation

Echoing findings from previous research see [2], the deep-seated concerns around imbalances in decision-making power, autonomy and representation among cross-sectoral actors (particularly animal and public health functionaries) were widely discussed across interviews. On face value, whereas the dual zoonoses governance structure (revolving around animal health and human health) affords autonomy and parity in terms of decision-making authority, in practice, the picture is much more complex. In this regard, it emerged from the interviews that issues around longstanding disparities in decision-making power and resource control characterised recent OH initiatives, impacting adversely on the level of support and/or participation by often self-perceived ‘fringe’ actors. State and district level animal health actors, for example, were particularly discontented about the alleged entrenched hegemony or superiority complex of their human health counterparts as some raised concerns about the disproportional representation (even in budgetary allocations) and limited mutuality in decision-making platforms. Two critical views expressed by a state-level actor and an expert from the animal health sector are instructive:

*“We are nowhere in the say in terms of strategic OH policy decision-making happening in the AC rooms. They [human health actors] will not be ready to listen to you. If you go to them and you tell them this is what we need to do, they will not at all be willing to listen to you. The policy matters have been directed towards protection of mankind. We are working to protect humankind, aren’t we [sic]? And the animal health side is completely side-lined.”* (Interview 8, Animal Health-State level).

“*Everything is diverted towards ICMR institutions and veterinary institutions feel side-lined in the OH engagement process. If you just look at the history of funding, for example, you know it is all towards humans only. Why is this disparity? When we talk about One Health, where do you want us [animal health institutions] to be? We are nowhere in the say to influence decision-making. Do you want us to do only the dirty work?*” (Interview 5, Animal Health-State level).

Advocating the need for strengthening collective decision-making through the establishment of a community of practice on zoonoses surveillance and control (leveraging lessons from the Covid-19 experience), some human health affiliates conceded that current OH-oriented activities were largely human health centred with limited avenues for non-health actors (e.g. wildlife, forestry departments) to engage. One human health actor commented that, “*everyone behaves as if only the* [human] *health affiliated departments have to do everything and they* [animal health, forestry and wildlife departments] *do not have any responsibility”* (Interview 11, Public Health-District level). From a non-health perspective, two district-level informants (from the forestry and wildlife departments) mentioned that the skewed biomedical focus of supposed cross-sector OH initiatives conveyed the understanding that their (i.e. non-health agencies) contributions are peripheral. A state-level actor from the animal health sector seemingly validated the above observation positing, *“they [human health actors] will not be at all ready to listen to you. If you go to them and you tell them this is what we need to do, they will not at all be willing to listen to you. They won’t take our ideas!*” (Interview 8, Animal Health-State level). To the extent that the foregoing holds true, it is not surprising human health actors dictated decisions and actions emanating from supposedly cross-sector OH committees. Speaking to the ‘skewed’ human health focus of extant OH research initiatives, a public health expert for instance explained that funder priorities coupled with the tedium of identifying relevant experts (in other ‘fringe’ on-health departments) has meant that organisers of cross-sector OH-related initiatives often deferred to a less complicated option of choosing/inviting ‘known’ experts to participate. Indeed, a number of animal health affiliates claimed that the “domination” of human health actors in extant cross-sector OH initiatives operated as a ‘red-tape’ associated with engagement with non-human health actors, even in instance where such collaborations are sanctioned from the top.

#### Trust and mutuality

A consensus across the interviews was the critical role long-term stakeholder relationships (i.e. formal and informal connections between different actors) and mutuality plays in overcoming communication asymmetries and negotiating ‘win–win’ collaborative outcomes. Reminiscing on the shared learning and value derived from the Covid-19 induced collaborations, several interviewees (mostly state and district level actors) noted how they had leveraged both extant institutional and even (informal) personal networks to facilitate rapid cross-departmental decision-making/intervention as part of India’s pandemic response. A typical view by a state-level bureaucrat reflecting on how the maintenance of strong relationships at both the institutional and individual levels is an essential prerequisite in driving cross-sectoral collaborations is illuminating:

“*Strong relationships are critical in fostering partnerships between government agencies and without strong relationships it is difficult to sustain collaborations outside of disease outbreak emergencies. So, I think it’s more of getting a rapport between these different departments, once you develop that, I think it* [collaborations] *will become very streamlined. Covid-19 has given impetus to the need of the hour…*” (Interview 9, Public Health-State level).

Corroborating the above observation, a high ranking national-level bureaucrat argued that some state health departments (across the human and animal health sectors – e.g. Tamil Nadu and Kerala) in complementing strategic national-level efforts are spending significant amount of time and resources to build relations of trust and credibility (through joint meetings/programmes) as a precursor to driving substantive OH-centred collaborations [42]. Among other things, the said official, for instance, had this to say about the current state of play:

*“There are some agencies/departments [health-centred ones] that are very comfortable working with each other [because historical ties and/or commonality of focus]. So, if they can earn the trust of the other [non-health] departments, then they can bridge the gap between health and non-health departments in fostering typical OH collaboration for zoonoses management. To put it in a nice way, existing OH-type collaborations are largely based on whom you know, that is organisations/departments that are comfortable working with each other”* (Interview 12, Public Health-National level).

On the cross-sectoral interplay in zoonoses surveillance and control, collaborative outcomes were heavily influenced by who is at the helm of the decision-making in respective departments and their personal predisposition to cross-sector engagement. As one national-level informant explained, *“over time I have observed that persons who have been working on particular zoonotic diseases for a long time are more open and supportive of cross-department collaborations. Controlling the particular disease becomes a priority for them so you can gauge their interest in facilitating work* [i.e. collaborative activities] *when you speak to them*” (Interview 5, Animal Health-State level). It thus follows that amidst the multiple barriers to cross-sectoral engagement (e.g. conflicting priorities, time constraints etc.), actors with high social capital in practice tend to have the capacity to wade through these challenges and marshalling tangible institutional support (including alignment of key institutional policies) towards favouring certain cross-sector collaborations. In this sense, the degree of social capital of departmental leads (responsible for championing/managing cross-sector engagement) determined their level of exposure to collaborative opportunities, particularly outside of disease outbreak emergencies, which suggests that individuals who are less pro-active and/or have less social capital were somewhat isolated from key social networks within which collaborative efforts often emerged. Nevertheless, some informants expressed some concern about overly relying on informal collaborative networks (in steering cross-sector partnerships) not backed by explicit formal institutional agreements given the propensity of loss of institutional memory through staff rotations/transfers, departmental leadership changes etc. A state-level informant commented:

*“There is a lot of work to be done [to sustain cross-sector collaborations], people change, and systems change. But then how do we retain institutional memory and how do we keep record of these key persons who are working in different departments? We often do this on informal basis which means no clear institutional guidelines for capturing these relations[networks]”* (Interview 19, Public Health-State level).

To buttress this assertion, another state level respondent intimated, *“officers keep changing at every place, every new officer will have to sensitized on extant cross-departmental relationships which is difficult if fostered informally”* (Interview 20, Animal Health-State level). The above highlights the view that collaborations are not neutral elements but socio-political formations in that the level of social capital of individual actors is a critical determinant of the quality and scope of collaborative networks.

## Discussion

Despite the widespread endorsement of the OH paradigm for improving zoonoses surveillance and control, there is paucity of empirical research on the contextual dynamics of cross-sectoral collaborations, particularly stakeholders’ perceptions of collaboration, how different collaborations emerge and drivers of stakeholder interactions [3, 5, 41]. This is critical given that stakeholder positionality and influence can affect the formation of collaborative networks and associated outcomes [21]. We examined the stakeholder ecosystem for zoonoses governance to better understand the ‘ties that bind’ and factors that modulate stakeholders’ decision-making and priorities concerning stakeholder engagement. In comparing our findings to the existing OH literature, we note that the operationalisation of OH initiatives in the global South has at best been limited due to the interplay of governance and contextual factors that are still poorly understood [1, 3, 21]. Whereas all stakeholder groups emphasised the importance of cross-sectoral collaboration in tackling the zoonoses challenge, there was shared understanding that the collaboration structure alone will not be able to achieve the aims of collaboration. Importantly, we unravelled the complexity of stakeholder networking highlighting the interaction of different contextual and perceived factors that determine collaborative outcomes across the human health, animal health and ecosystem interface in India. In this respect, stakeholder involvement in OH initiatives tended to be determined by time commitment, institutional bureaucracies and representation, supporting the argument that generating the requisite institutional incentives remain critical to drive collaborations [17, 41].

Reflecting a common critique about the abstractness of the OH concept [6], our findings highlight that the majority of extant OH networks linked to zoonoses prevention and control (which emanated from time-bound research projects) were dominated by research-oriented institutions (e.g. ICMR and ICAR affiliated research institutes) with limited scope for practical operationalisation in the long run. OH implementation is largely viewed from an academic/research dominant lens, which may partly account for the limited institutional incentives to collaborate and the lack of task allocation outside of outbreak scenarios. Whereas some scholars [4] have argued for a centralised administrative platform for OH operationalisation, the ill-defined guidelines can lead to stakeholder tensions particularly around expectations on roles and responsibilities in light of competing priorities/agendas [2, 21]. Indeed, a pre-defined centralised OH platforms (e.g. National Standing Committee on Zoonoses (NSCZ)) alone risk ‘fossiling’ the institutionalisation process such that there is little room for innovation and/or evolution (at the state level) especially in federated governance systems like India. In this sense, flexible multi-level OH committees that are legally mandated and tailored to sectoral (beyond Public Health) and/or state level preferences remain necessary in the current zoonoses governance landscape in India [2, 43].

To successfully implement proposed flexible, multi-level OH platforms, strategic investment in integrative science-policy interfaces (e.g. fostering dialogue between key policymaking and research institutes, OH departmental champions) remain paramount in strengthening trust-based relationships and mutuality that can facilitate exchange of knowledge and information for improved zoonoses surveillance and control. As evidenced in the interviews, in spite of institutional barriers (e.g. competing interests, distrust between actors, departmental bureaucracy), responsible actors with high social capital managed to circumvent them leveraging professional goodwill and informal networks to initiate and drive collaborations [1, 2, 5]. This lends empirical credence to the observation that decision-making and operationalisation of cross-sector OH networks/initiatives (for zoonoses prevention and control) cannot be divorced from the local institutional politics and socio-cultural context [1, 44]. As Abbas et al. [1] concluded that spontaneous cross-sectoral collaborations are complex enterprises resulting from the convergence of key externalities that produces a catalytic effect spurring “in-organic” coalescing of hitherto non-collaborating actors during disease outbreak emergencies.

The study has several policy implications. First, to the best of our knowledge, we believe this is the first multi-scale mapping of OH stakeholders and their interactions with respect to zoonoses governance in India at least in the published literature. This integrative analysis affords a better understanding of the dynamics of OH networks building on prior studies see [2, 4, 5]. Our qualitative synthesis at multiple levels allowed us to theorise on the drivers of cross-sector collaborations, highlighting the inherent barriers and opportunities which provides an entry point for stakeholder engagement on the incentive-based arrangements needed to stimulate greater cross-sector engagement [2, 22]. Second, the limited involvement of forestry and wildlife sectors in zoonoses-related OH initiatives has far-reaching implications for policymaking and funding mechanisms that could incentivise greater participation in zoonoses prevention/preparedness and control. Though conflicting actor priorities can be aligned by drift towards shared goals [2, 45], our findings highlight other modes of incentivising cross-sector engagement. Aside from principal actors negotiating common ground in some instances, they also capitalised on political/institutional pressure to subtly ‘manipulate’ their subordinates as a way of fostering cross-sector engagement, especially in instances when the institutional and political stakes are high. In such instances, ground level implementing actors are implicitly confronted with the choice of prioritising collaborations (at the expense of competing departmental priorities) or risk backlash for non-compliance with sanctioned top-down directives. This finding provides a fresh perspective on how political capital can be leveraged as means of temporary brokerage for cross-sector engagement in the face of competing stakeholder priorities/agendas in highly bureaucratic settings. However, a key sticking point is the sustenance of such politically induced collaborative engagements over time especially in the absence of a formalised OH strategy. Further scrutiny of how socio-political capital is leveraged in fostering cross-institutional engagement on zoonoses governance and associated drawbacks in different settings will provide additional insights on navigating contextual barriers given a complete overhaul of extant institutional structures remain highly unlikely [4, 21]. The Covid-19 induced collaborations are clear testaments of how politically conditioned cross-sector collaborations could hold sway in crafting collaborative arrangements such that they overcome institutional complexities and engender appropriate incentive structures.

As with any empirical study, our work is not without limitations. First of all, we did not include perspectives of non-state actors (including international organisations) who are involved in OH initiatives linked to zoonoses surveillance and control in India. Given our focus on interplay between state-managed institutions with direct mandates on zoonoses prevention and control, we felt that non-governmental stakeholders were out of scope but propose their inclusion in future studies for a more complete picture of the spectrum of OH-related collaborative networks for zoonoses governance in India. Moreover, the groundswell of externally sponsored OH initiatives in the global South implies that a systematic exploration of the role of non-state actors in the local and international spheres remain paramount. This could allow for nuanced interrogation of the financial and non-financial incentives driving externally sponsored OH initiatives and the variations in disease prioritisations across different socio-political and epidemiological contexts [3, 39]. Furthermore, while we mapped extant disease-specific OH initiatives in India, we recognise that the fluidity of the policy landscape and the geographical variability in disease endemicity suggest that further in-depth disease specific explanations of organisational networks and ties in outbreak and non-outbreak scenarios at granular (i.e. district and sub-district) scale could be a fruitful line of enquiry. Moreover, while we interviewed stakeholders across the spectrum of zoonoses prevention and control, we had relatively small number of interviewees, disproportionately skewed in favour public health actors. This is not surprising given that the predominance of public health actors in zoonoses governance and by extension OH policy discussions [2, 3]. Nevertheless, the careful cross-sector stakeholder recruitment ensured that the perspectives of otherwise underrepresented groups on OH-related initiatives linked to zoonoses governance were captured. Finally, longitudinal studies on exemplar One Health initiatives and stakeholder preferences and changes in engagement over time could offer lessons on mechanisms sustaining OH collaborations in the long term and the channels of funding mechanisms. We also encourage similar research in other global South contexts on OH stakeholder networks for zoonoses prevention/preparedness and control to validate our findings.

## Conclusion

As OH operationalisation continues to gain momentum across the global South as a plausible pathway for improved cross-sectoral engagement for zoonoses prevention and control, there are increasing calls to better understand the stakeholder and local governance contexts that determine collaborative outcomes [3, 21, 22]. India is no exception given the groundswell of OH-related initiatives aimed at strengthening local health systems for better prevention and control of zoonoses [29, 46]. This study characterised the OH stakeholder landscape identifying the state institutions responsible for disease outbreak prevention and control, their interrelationships and factors that enhance or impede cross-sector collaborations. Building on the existing OH scholarship [2, 4, 28], our study shows how the local socio-political and institutional contexts interact to modulate how and when collaborations occur (or not), the associated contingencies and stakeholder innovations in circumventing existing barriers to cross-sector collaborations. Our main message is that cross-sector collaborations are by-products of intricate political and institutional tinkering as long as individual stakeholder and institutional interests converge. To strengthen the ‘ties that bind’, these dynamics must be embraced to embed meaningful and sustainable collaborations in different local socio-political and administrative contexts.

## Data Availability

Given the richness of the qualitative data and the potential for identifying individual human participants and violating confidentiality of the key informants who participated in the semi-structured interviews, our study dataset will not be shared openly but may be available upon specific request. Researchers wishing to access the dataset used in this study should contact the UK Centre for Ecology & Hydrology Institutional Data Access contact via Dr Semeena Shamsudheen (email: semval@ceh.ac.uk).

## Abbreviations

AWBI: Animal Welfare Board
COHZTD: Consortium for One Health to Address Zoonotic and Transboundary Diseases
CZA: Central Zoo Authority
DAHD: Animal Husbandry Department
DBT: Department of Biotechnology
DST: Department of Science and Technology
FAO: Food and Agricultural Organisation
GOI: Government of India
HPAI: Highly Pathogenic Avian Influenza
ICAR: Indian Council for Agricultural Research
ICMR: Indian Council for Medical Research
IDSP: Integrated Disease Surveillance Project
IMTF: Inter-Ministerial Task Force
JMG: Joint Monitoring Group
KFD: Kyasanur Forest Disease
LMIC: Low/Lower Middle Income Country
MoH&FW: Ministry of Health and Family Welfare
MoA&FW: Ministry of Agriculture and Farmers Welfare
MoEF&C: Ministry of Environment, Forests and Climate Change
NADRES: National Animal Disease Referral Expert System
NCDC: National Centre for Disease Control
NGO: Non-governmental Organisation
NLM: National Livestock Mission
NIE: National Institute of Epidemiology
NDDB: National Dairy Development Board
NIMR: National Institute for Malaria Research
NIVEDI: National Institute of Veterinary Epidemiology and Disease Informatics
NSCZ: National Standing Committee on Zoonoses
NVBDCP: National Vector Borne Disease Control Programme
OH: One Health
OIE: Office International des Epizooties (World Animal Health Organisation)
RCZI: Roadmap to Combat Zoonoses in India
R&D: Research and Development
SARS: Severe Acute Respiratory Syndrome
SDG: Sustainable Development Goals; UN-REDD: United Nations Reducing Emissions from Deforestation and Forest Degradation Programme
VCI: Veterinary Council of India
WD: Wildlife Department
WII: Wildlife Institute of India
WHO: World Health Organisation
